# A Community-Based Construct Method for an Inter-Satellite Communication Network of Satellite Swarm

**DOI:** 10.3390/e25010121

**Published:** 2023-01-06

**Authors:** Weicheng Lun, Qun Li, Can Zhang, Zhi Zhu

**Affiliations:** College of Systems Engineering, National University of Defense Technology, Changsha 410073, China

**Keywords:** inter-satellite communication network of satellite swarm, complex network, community, structural entropy, dynamic path

## Abstract

An inter-satellite communication network of satellite swarm (ICNS) is created if the members of a satellite swarm communicate with each other via inter-satellite links (ISLs). ICNS can be constructed using the theory of complex networks. A link community is defined as two satellites between which the ISL has been established. The satellite swarm, whose members have not established ISLs, is modeled as a pre-link network (PLN). The edge of a PLN is described as a candidate for the link community. Consequently, an ICNS can be constructed by collecting combinations of candidates for link communities (CCLC) based on PLN and then by selecting one of these. An algorithm is designed to take a sample of all CCLCs. A new structural entropy of networks is developed to evaluate a CCLC. The CCLC with a maximum structural entropy in the CCLC sample will be selected to become the edge set of the ICNS. An improvement method was proposed to ensure that the ICNS remains a dynamic connected network by preventing each satellite from establishing an ISL with the same satellite. The simulations demonstrated that the proposed method outperformed the benchmark methods, and it is necessary to adopt the improvement method.

## 1. Introduction

Many satellites are supposed to be organized as a satellite swarm to complete space tasks because the swarm intelligence will emerge with multiple autonomous satellite clustering. The members of the satellite swarm, i.e., the satellites, must communicate with each other via inter-satellite links (ISLs), which is the precondition for the satellite swarm’s operations. The satellite swarm creates an inter-satellite communication network of satellite swarm (ICNS) when its members are connected by ISLs.

An ICNS is a type of satellite network that treats satellites as its nodes and ISLs as its edges. Related work on the construction of a satellite network always focuses on transforming it into an optimization problem concerning ISL assignment or topology design. Ren et al. [1] modeled the ISL assignment problem as the decision optimization of the ISL selection action sequence, in which reinforcement learning is suitable for obtaining the solution. Yan et al. [2] transformed the ISL assignment scheduling problem in global navigation satellite systems (GNSS) into a multi-objective optimization problem and solved it using non-dominated sorting genetic algorithm II. Sun et al. [3] proposed assignment algorithms of time synchronization links based on satellite layers and ranging links combining satellite selection and weighting. Yan et al. [4] modeled the ISL assignment problem in GNSS as an integer programming problem and designed a rolling weight-matching method (RWM) and a rolling weight-matching method with weight enhancement (RWMWE) to solve it. Liu et al. [5] used a multi-objective simulated annealing algorithm and a two-step algorithm to assign laser ISL and radio ISL, respectively. Yan et al. [6] applied integer linear programming to solve the topology design problem in the context of GNSS, considering both ranging and communication performance. Zeng et al. [7] proposed multi-objective discrete binary particle swarm optimization to optimize the topology of optical inter-satellite links in GNSS. These studies prefer to apply intelligent optimization methods, which has produced many positive results, yet there are also some noteworthy features in these studies, such as the dependence of prior data, a large amount of calculation, and a high number of experiments.

Meanwhile, a satellite network belongs to the complex network, which is well-known for its complexity of nodes, structures, and evolution [8]. Some studies exist that have researched satellite networks through the theory of complex networks. Wang et al. [9] investigated the networking feasibility of quantum key distribution constellation networks. Sun et al. [10] selected the super satellite nodes according to the important coefficient of the satellite node. Liu et al. [11] presented a method based on a space–time evolving graph to estimate the required wavelengths in a dynamic optical satellite network. Lu et al. [12] designed a framework based on topological dynamics to evaluate the structural performance of satellite networks. Chen et al. [13] proposed a topological dynamics shielding method for LEO satellite networks by establishing a static virtual network. These studies employed the theory of complex networks to analyze the functions or performances of satellite networks, but they ignored the construction of these satellite networks.

It is obvious that combining the construction of a satellite network and the theory of complex networks has attracted less attention, to date. In fact, a satellite network deserves to be constructed with the theory of complex networks because it is actually a type of complex network. The method to construct a satellite network using the theory of complex networks has the following three advantages:

1. There is no limit on the type of satellites or constellations, so it is highly adaptable, which will help standardize a set of norms in the satellite network model;
2. It is based on the interaction of nodes, i.e., satellites, which can describe clearly how the structure of a satellite network affects its properties, behaviors, and performance;
3. It is able to interpret satellite networks in the way of systems or systems-of-systems and to reveal the nonlinearity of satellite networks.

Therefore, this paper aimed to apply the method mentioned above to study ICNS and offers a new course of research on satellite networks and the satellite swarm.

ICNS was the subject of this study, and the remainder of this paper is structured as follows: In Section 2, we described the process of modeling a satellite swarm as a complex network through the theory of complex networks and the necessity of this method. In Section 3, we introduced the method to construct an ICNS, which is based on the community structure and the structural entropy of complex networks. In Section 4, we put forward an optimization method to ensure that the ICNS remains as a dynamic connected network constantly. In Section 5, we designed some simulations to test and verify the performance of these proposed methods and analyzed their outcomes.

## 2. Complex Network Models of the Satellite Swarm

### 2.1. Introduction of Slots

**Figure 1:**
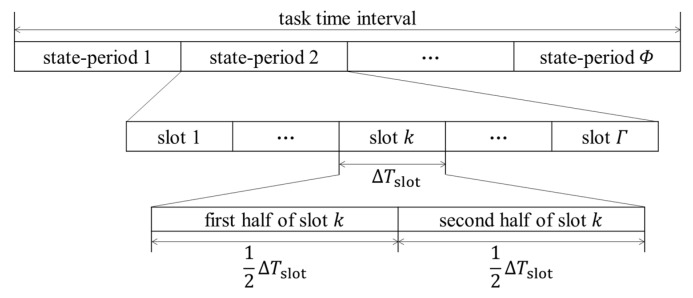
Division of a task time interval through FSA.

The communication task of a satellite swarm, such as transmitting data and orders, are always time-sensitive, which means there is a task time interval during which the task must be fulfilled. It is unnecessary to worry about which ISLs are established in the satellite swarm every second because there is a better way to cope with the establishment of ISLs during the task time interval. That method is the finite state automata (FSA) [14,15], which can save computing and storage resources and improve the management simplicity of link planning [2]. As Figure 1 shows, the task time interval is divided into Φ equal parts ($\Phi \in N_{+}$), which are called state periods; then, each state period is further divided into Γ equal parts (1≤k≤Γ), which are called slots. Each slot corresponds to an FSA state indicating which ISLs are established. As each slot will occur in the cycle, we only need to focus on these Γ FSA states. The topology of an ICNS is believed to remain unchanged in each slot. 

The length of a slot is denoted by $\Delta T_{\mathrm{slot}}$. All temporal concepts were counted by the slot in this paper unless there is a special explanation.

### 2.2. Introduction of ICNS

**Figure 2:**
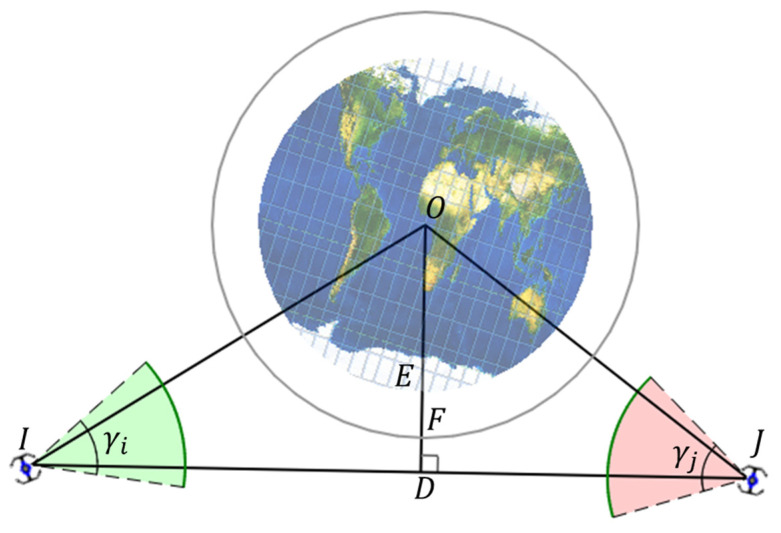
Conditions of establishing an ISL.

The members of a satellite swarm are denoted by $V=\{v_{i}\}$, where $v_{i}$ represents the *i*-th satellite. The cardinality of V is designated by Θ (i.e., |V|=Θ). If $v_{i}$ and $v_{j}$ need to establish ISL within slot k, two conditions must be met throughout this slot:

1 The distance between the Earth’s core and the line from $v_{i}$ to $v_{j}$ (|OD| in Figure 2) is greater than the sum of the Earth’s radius $v_{j}$ (|OE| in Figure 2) and the height of the ionosphere(|EF| in Figure 2), as is shown in Equation (1):

(1) |OD|>|OE|+|EF|

2 The elevation angle from $v_{i}$ to $v_{j}$ (∠OIJ in Figure 2) is in the onboard antenna scanning range of $v_{i}$ ($\gamma_{i}$ in Figure 2); meanwhile, the elevation angle from $v_{j}$ to $v_{i}$ (∠OJI in Figure 2) is in the onboard antenna scanning range of $v_{j}$ ($\gamma_{j}$ in Figure 2), as is shown in Equation (2):

(2) $$\{\begin{array}{c} ∠OIJ<\gamma_{i}\\ ∠OJI<\gamma_{j} \end{array}$$

Once ISLs are established in a satellite swarm, an ICNS is created. According to the notation of the graph theory, the ICNS that is created during slot k can be modeled as a graph $G_{k}=(V_{k},E_{k})$, where $V_{k}=\{v_{i}\}$ is the node set and $E_{k}=\{e_{ij}|v_{i},v_{j}\in V,i<j\}$ is the edge set. Herein, nodes are equivalent to satellites and edges are equivalent to ISLs. Thus, $v_{i}$ is not only the *i*-th satellite but also the *i*-th node. Furthermore, $e_{ij}$ is not only the edge connecting the *i*-th node and the *j*-th node but also the ISL between the *i*-th satellite and the *j*-th satellite.

In this paper, $e_{ij}$ does not indicate the direction of ISL but only means that there is an ISL between $v_{i}$ and $v_{j}$. The satellite with a smaller number $v_{i}$ is called the left satellite while the satellite with a bigger number $v_{j}$ is called the right satellite. An ICNS is not an undirected graph, and it is not a simple directed graph. In fact, there is two-way alternate communication between two satellites connected with an ISL, that is, the direction of ISL is from the left satellite to the right satellite during the former half slot and from right the satellite to the left satellite during the former half slot. Thus, the ICNS is a special directed graph whose edges have changeable directions.

The edge set $E_{k}$ is also a set of ISLs that are established during slot k. As a satellite is only allowed to establish one ISL with another satellite within a slot [6], $E_{k}$ has $\frac{1}{2}\Theta$ elements at most. The difference between these ICNSs for various slots only concerns ISLs rather than satellites, according to [12]. Therefore, the ICNS for slot k can be further denoted by $G_{k}=(V,E_{k})$. As a result, the construction of ICNS can be reducible to the construction of the edge set of the ICNS for each slot. Since each satellite establishes at most one ISL in any slot, no two edges in an ICNS have a vertex in common. Hence, the ICNS is a disconnected graph at any time.

### 2.3. Pre-Link Network

If there is no ISL to establish in a satellite swarm, the ICNS will not be created. Under the circumstances, the satellite swarm can be modeled as a pre-link network (PLN). The PLN is an imaginary network that plays a vital role in constructing an ICNS. The PLN modeled to construct $G_{k}$ is denoted by ${\tilde{G}}_{k}=(V,{\tilde{E}}_{k})$, where V is the node set and ${\tilde{E}}_{k}=\{{\tilde{e}}_{ij}|v_{i},v_{j}\in V,i<j\}$ is the edge set. The edge ${\tilde{e}}_{ij}$ means the couple of $v_{i}$ and $v_{j}$ between which an ISL can be established during slot k rather than a real ISL. In additionrather than a real ISL. In addition, there is $|{\tilde{E}}_{k}|\leq \left(\begin{array}{c} \Theta \\ 2 \end{array}\right)$.

Although the satellite swarm actually creates an ICNS by establishing some ISLs, we can give a new explanation for the construction of an ICNS, which is based on the theory of complex networks: we first tease out all satellite pairs that meet the condition for establishing ISLs and create a PLN through them; then, we convert the PLN, which has more edges, into an ICNS, which has fewer edges, by selecting some particular edges.

## 3. Community-Based Construct Method of an ICNS 

### 3.1. Link Community

Communities in networks are groups of nodes. There are dense connections in the communities and sparser connections between the communities [16]. As a satellite is only allowed to establish one ISL with another satellite within a slot, an ICNS is composed of multiple satellite pairs, within which there are ISLs yet between which there are no ISLs. These satellite pairs can be treated as communities, which are called link communities. The link community has two essential features: (1) one link community contains two and only two nodes, and (2) there is no edge between any two link communities. We propose that the link community is equivalent to the ISL, so the link community that consists of $v_{i}$ and $v_{j}$ is also denoted by $e_{ij}$. The edge ${\tilde{e}}_{ij}$ in the PLN represents a candidate for the link community (CLC) $e_{ij}$ accordingly.

In fact, the ICNS is a network that is composed of θ link communities if there are θ satellite pairs that have established ISLs ($\theta \leq \frac{1}{2}\Theta$). The combination of candidates for link communities (CCLC) is defined as a special subset of the edge set of a PLN in which no two members, i.e., candidates for link communities, have a node in common. Hence, $G_{k}$ can be constructed by collecting CCLCs based on ${\tilde{G}}_{k}$ and by choosing one of them to become $E_{k}$. 

We then introduced three fundamental matrices of ${\tilde{G}}_{k}$, which are vital to construct ICNS. They are the adjacency matrix $A_{k}={\left(a_{ij}^{\left[k\right]}\right)}_{\Theta \times \Theta }$, degree matrix $D_{k}={\left(d_{ij}^{\left[k\right]}\right)}_{\Theta \times \Theta }$, and weight matrix $W_{k}={\left(w_{ij}^{\left[k\right]}\right)}_{\Theta \times \Theta }$. The element $a_{ij}^{\left[k\right]}$ of $A_{k}$ is defined by Equation (3).

(3) $$a_{ij}^{\left[k\right]}=\{\begin{array}{ll} 1, & {\tilde{e}}_{ij}\in {\tilde{E}}_{k}\\ 0, & {\tilde{e}}_{ij}\notin {\tilde{E}}_{k}\;\mathrm{or}\;i=j \end{array}$$

The element $d_{ij}^{\left[k\right]}$ of $D_{k}$ is defined by Equation (4).

(4) $$d_{ij}^{\left[k\right]}=\{\begin{array}{ll} \mathrm{degree}\;\mathrm{of}\;v_{i}\;\mathrm{in}\;{\tilde{G}}_{k}, & i=j\\ 0, & \mathrm{others} \end{array}$$

The element $w_{ij}^{\left[k\right]}$ of $W_{k}$ is defined by Equation (5).

(5) $$w_{ij}^{\left[k\right]}=\{\begin{array}{ll} \mathrm{weight}\;\mathrm{of}\;{\tilde{e}}_{ij}, & a_{ij}^{\left[k\right]}=1\\ 0, & \mathrm{others} \end{array}$$

There are two steps to obtain $W_{k}$. Firstly, we establish a distance matrix $R={\left(r_{ij}^{\left[k\right]}\right)}_{\Theta \times \Theta }$ whose element $r_{ij}^{\left[k\right]}$ is defined by (6).

(6) $$r_{ij}^{\left[k\right]}=\{\begin{array}{ll} \mathrm{distance}\;\mathrm{between}\;v_{i}\;\mathrm{and}\;v_{j}, & a_{ij}^{\left[k\right]}=1\\ 0, & \mathrm{others} \end{array}$$

Secondly, we normalizes $r_{ij}^{\left[k\right]}$ as (7), so that $w_{ij}^{\left[k\right]}$ is obtained.

(7) $$w_{ij}^{\left[k\right]}=\{\begin{array}{ll} \frac{\underset{a_{\lambda \mu }^{\left[k\right]}=1}{\min}r_{\lambda \mu }^{\left[k\right]}}{r_{ij}^{\left[k\right]}}, & a_{ij}^{\left[k\right]}=1\\ 0, & \mathrm{others} \end{array}$$

In this paper, $d_{ii}^{\left[k\right]}$ denotes the degree of $v_{i}$ in ${\tilde{G}}_{k}$ and $w_{ij}^{\left[k\right]}$ denotes the weight of ${\tilde{e}}_{ij}$ in ${\tilde{G}}_{k}$.

### 3.2. Algorithm to Sample a CCLC

A CCLC is a matching of a PLN according to graph theory, since the CCLC is a set of edges in the PLN that are pairwise disjointed. If the cardinality of a CCLC is $\frac{1}{2}\Theta$, the CCLC is a perfect matching of the PLN. As the enumeration problem for perfect matchings in general graphs is NP-hard [17], it is almost impossible to count the number of all CCLCs. In addition, if there were a large number of satellites, the number of accessible available CCLCs must be astronomical. Consequently, the enumeration of CCLCs is unavailable.

Therefore, we attempted to collect a sample of all CCLCs. The sample was required to cover all of ${\tilde{E}}_{k}$, which ensured the representativeness of the sample. The sample of all CCLCs that was used to construct $G_{k}$ is designated by ${ℳ}_{k}=\{M_{k}(\varepsilon )\}$, where $M_{k}(\varepsilon )$ denotes the *ε-*th CCLC in ${ℳ}_{k}$. ${ℳ}_{k}$ can be obtained by Algorithm 1.
**Algorithm 1.** Sample algorithm for CCLC.**Input:** Adjacency matrix $A_{k}$ of ${\tilde{G}}_{k}$ **Output:** A subset of all CCLCs ${ℳ}_{k}$
1:  Establish an empty set of CCLC ${ℳ}_{k}=\{\}$
**and**
ε=1
 2:  **for** i=1
**to**
Θ
**do**
 3:   **for**
j=i+1
**to** Θ
**do**
 4:      **if**
$a_{ij}^{\left[k\right]}=1$
**and**
${\tilde{e}}_{ij}\notin E^{\prime }$
**then**
 5:     Establish a set of visited nodes $V^{\prime }=\{v_{i},v_{j}\}$
**and** a set of visited edges         $E^{\prime }=\{{\tilde{e}}_{ij}\}$
 6:     **for**
y=1
**to**
Θ
**do**
 7:       Establish a CCLC $M_{k}(\varepsilon )=\{{\tilde{e}}_{ij}\}$
 8:       **for**
λ=1
**to**
Θ
**do**
 9:         **for**
μ=Θ
**to**
1
**do**
 10:           **if**
$v_{\lambda },v_{\mu }\notin V^{\prime }$, ${\tilde{e}}_{\lambda \mu }\notin E^{\prime }$, **and**
$a_{\lambda \mu }^{\left[k\right]}=1$
**then**
 11:            $M_{k}(\varepsilon )\leftarrow M_{k}(\varepsilon )\cup \{{\tilde{e}}_{\lambda \mu }\}$, $V^{\prime }\leftarrow V^{\prime }\cup \{v_{\lambda },v_{\mu }\}$, $E^{\prime }\leftarrow E^{\prime }\cup \{{\tilde{e}}_{\lambda \mu }\}$
 12:            **break**

13:           **end if**

14:         **end for**

15:       **end for**

16:       **if**
$M_{k}(\varepsilon )\notin {ℳ}_{k}$
**then**
 17:           ${ℳ}_{k}\leftarrow {ℳ}_{k}\cup \{M_{k}(\varepsilon )\}$, ε←ε+1
 18:        **end if**

19:      **end for**

20:     **end if**

21:    **end for**

22: **end for**

Not all CCLCs in ${ℳ}_{k}$ obtained by Algorithm 1 must have θ elements, so ${ℳ}_{k}$ needs to be modified. If $\theta =\frac{1}{2}\Theta$, the CCLCs that are less than $\frac{1}{2}\Theta$ should be removed from ${ℳ}_{k}$. If $\theta <\frac{1}{2}\Theta$, the CCLCs whose cardinalities are not equal to θ should be removed from ${ℳ}_{k}$; however, the subsets with θ elements of the CCLCs whose cardinalities are more than θ should be added into ${ℳ}_{k}$. We can apply the Tutte theorem [18] to determine the existence of a perfect matching in a PLN; if a perfect matching exists, θ should be $\frac{1}{2}\Theta$ to maximize the number of ISLs. 

### 3.3. Method of Selecting a CCLC According to Structural Entropy

Now that ${ℳ}_{k}$ has been obtained by Algorithm 1, the next step is to select a suitable CCLC from ${ℳ}_{k}$. We will introduce a method of selecting CCLC according to structural entropy in this section.

The structural entropy is a type of network entropy [19], which is a measure of the network’s heterogeneity [20]. The weaker the heterogeneity, the greater the entropy value [21]. A weaker heterogeneity means there are fewer differences between nodes of the network [22]. Nowadays, there are many kinds of structural entropies in the field of complex networks [19]. We borrowed the idea from the authors in [23], that is, considering both nodes and edges to define a new structural entropy.

We treat each CLC of $M_{k}(\varepsilon )$ as a node. We state that if ${\tilde{e}}_{ij}$ is the *ι*-th CLC in $M_{k}(\varepsilon )$, ${\tilde{e}}_{ij}$ can be denoted by $c_{\iota }$. We define the degree $d_{c}^{\left[\iota \right]}$ of $c_{\iota }$ as the normalized number of links incident to nodes in $c_{\iota }$:

(8) $$\begin{array}{c} d_{c}^{\left[\iota \right]}=\frac{d_{ii}^{\left[k\right]}+d_{jj}^{\left[k\right]}-2}{(\sum_{\lambda =1}^{\Theta }d_{\lambda \lambda }^{\left[k\right]})-2\theta } \end{array}$$

We define the weight $w_{c}^{\left[\iota \right]}$ of $c_{\iota }$ as the weight of ${\tilde{e}}_{ij}$, i.e., $w_{c}^{\left[\iota \right]}=w_{ij}^{\left[k\right]}$. Then, we define the importance coefficient $U_{\iota }$ of $c_{\iota }$ as the weighted sum of $d_{c}^{\left[\iota \right]}$ and $w_{c}^{\left[\iota \right]}$:

(9) $$U_{\iota }=\alpha d_{c}^{\left[\iota \right]}+\beta w_{c}^{\left[\iota \right]}$$

where α and β are weights and α+β=1. $U_{\iota }$ needs to be normalized as follows:

(10) $${\bar{U}}_{\iota }=\frac{U_{\iota }}{\sum_{\upsilon =1}^{\theta }U_{\upsilon }}=\frac{\alpha d_{c}^{\left[\iota \right]}+\beta w_{c}^{\left[\iota \right]}}{\sum_{\upsilon =1}^{\theta }\left(\alpha d_{c}^{\left[\upsilon \right]}+\beta w_{c}^{\left[\upsilon \right]}\right)}=\frac{\frac{\alpha }{\beta }d_{c}^{\left[\iota \right]}+w_{c}^{\left[\iota \right]}}{\sum_{\upsilon =1}^{\theta }\left(\frac{\alpha }{\beta }d_{c}^{\left[\upsilon \right]}+w_{c}^{\left[\upsilon \right]}\right)}$$

We believe that $d_{c}^{\left[\iota \right]}$ is as important as $w_{c}^{\left[\iota \right]}$, i.e., α=β. Thus, (10) is transformed into

(11) $${\bar{U}}_{\iota }=\frac{d_{c}^{\left[\iota \right]}+w_{c}^{\left[\iota \right]}}{\sum_{\upsilon =1}^{\theta }\left(d_{c}^{\left[\upsilon \right]}+w_{c}^{\left[\upsilon \right]}\right)}$$

Finally, we give the formula of the structural entropy $H_{\varepsilon }^{\left[k\right]}$ of $M_{k}(\varepsilon )$ with θ CLCs:

(12) $$H_{\varepsilon }^{\left[k\right]}=-\sum_{\iota =1}^{\theta }{\bar{U}}_{\iota }\ln{\bar{U}}_{\iota }$$

ICNS should have lower heterogeneity to help satellites make full use of their autonomy, which will promote the decentralization and swarm intelligence of a satellite swarm. Therefore, the maximum structural-entropy CCLC (MSE-CCLC) should be selected to become $E_{k}$, as shown in Equation (13):

(13) $$E_{k}=\{e_{ij}|{\tilde{e}}_{ij}\in M_{k}(\underset{\varepsilon }{\mathrm{argmax}}H_{\varepsilon }^{\left[k\right]})\}$$

## 4. Optimized Constructions of ICNS

### 4.1. ICNS for One State Period

Although the lengths of ISLs in the ICNS for one single slot (ICNS-OSS) will change over time, the type and number of ISLs remains stable throughout the slot. Thus, the ICNS for a single slot cannot be treated as a dynamic network. However, the ICNS for multiple slots is a typical dynamic network, in which both lengths and existences of the edges change over time. Obviously, an edge exists during some slots due to the establishment of the corresponding ISL, and it disappears during another slot due to the disconnection of the ISL. This section focuses on the ICNS for one state period (ICNS-OSP) and its optimization. An ICNS-OSP can be represented by a link matrix $X={\left(x_{ik}\right)}_{\Theta \times \Gamma }$. The element $x_{ki}$ of X is defined as shown in Equation (14).

(14) $$\begin{array}{c} x_{ik}=\{\begin{array}{ll} v_{j}, & \mathrm{there}\;\mathrm{is}\;\mathrm{an}\;\mathrm{ISL}\;\mathrm{between}\;v_{i}\;\mathrm{and}\;v_{j}\;\mathrm{during}\;\mathrm{slot}\;k\\ v_{i}, & v_{i}\;\mathrm{connects}\;\mathrm{with}\;\mathrm{no}\;\mathrm{satellite}\;\mathrm{during}\;\mathrm{slot}\;k \end{array} \end{array}$$

Some important sets with respect to the ICNS-OSP are defined as follows:

4. The set of selected CCLCs $S=\{{\bar{M}}_{k}|1\leq k\leq \Gamma \}$. The element ${\bar{M}}_{k}$ of S denotes the CCLC that is selected to become $E_{k}$. S is equivalent to X for they both represent the relationship between ISLs and slots in an ICNS-OSP.
5. The set of all ISLs $\hat{E}=E_{1}\cup \cdots \cup E_{\Gamma }$. $\hat{E}$ contains all the ISLs that have been established within one state period. The ISLs that are established within different slots but include the same satellites are the same.
6. The set of link objects $O_{i}=\{v_{j}|v_{j}\in V,e_{ij}\in \hat{E}\}$. $O_{i}$ contains all the satellites that have connected with $v_{i}$ via ISLs. The cardinality of $O_{i}$, i.e., $\left|O_{i}\right|$, is defined as the general degree of $v_{i}$, which represents the number of link objects of $v_{i}$.
7. The set of potential link objects ${\tilde{O}}_{i}=\{v_{j}|v_{j}\in V,{\tilde{e}}_{ij}\in {\tilde{E}}_{k},1\leq k\leq \Gamma \}$. ${\tilde{O}}_{i}$ contains all the satellites that meet the conditions to establish ISLs with $v_{i}$. The cardinality of ${\tilde{O}}_{i}$, i.e., $\left|{\tilde{O}}_{i}\right|$, represents the number of potential link objects of $v_{i}$.

### 4.2. Dynamic-Connected and Dynamic Paths

PLNs for different slots may have the same adjacency matrices and degree matrices, which may lead to the same sets of CCLCs. Although different slots can result in different weight matrices, it is possible to select the same CCLC according to structural entropy, that is, the MSE-CCLCs for different slots may be the same. In this situation, each satellite would keep establishing the ISL with the same satellite. As there is no edge between any two link communities, an ICNS-OSS must be non-connected. Consequently, if all satellites do not change their link objects over time, the ICNS-OSP will remain disconnected throughout the state period. In order to ensure effective communication in the satellite swarm, the ICNS-OSP must be dynamically connected, which requires that the dynamic paths between any two satellites are always available.

A dynamic path in ICNS is a space–time sequence of nodes that begins with the source node and ends with the destination node. The space–time sequence indicates that a dynamic path has both temporal length and spatial length. The temporal length is the number of slots that a dynamic path spans. The spatial length is the number of edges that are constituted of two neighboring nodes in a dynamic path. We define an S&D (the abbreviation of “source and destination”) as the ordered pair $\left(v_{i},v_{j}\right)$ that contains the source node $v_{i}$ and the destination node $v_{j}$. The dynamic path for an S&D $\left(v_{i},v_{j}\right)$ is written as follows:

(15) $$DP(v_{i},v_{j},k)=\{v_{i}^{\left[k\right]},\cdots ,v_{j}^{\left[k+p\right]}\}$$

where $v_{i}^{\left[k\right]}$ represents $v_{i}$ during slot k, and so on. There are two important parameters in (15): k is the slot in which the dynamic path is planned and it is called the plan slot (1≤k≤Γ), and k+p is the slot in which the dynamic path finishes and it is called the finish slot (p≥0). Consequently, p+1 represents the temporal length of $DP(v_{i},v_{j},k)$.

A dynamic path can be planned by the method of finding the earliest route from a source satellite to all the other satellites [24]. Given the slot k, if every S&D $\left(v_{i},v_{j}\right)$ has an existent $DP(v_{i},v_{j},k)$, the ICNS is believed to be dynamically connected from slot k to slot k+p. Under the circumstances, the maximum temporal length of all dynamic paths can be defined by dynamic connect duration (DCD), which means how long it takes an ICNS to achieve dynamic connectivity from slot k. However, if some S&D does not have a dynamic path, the ICNS will never be dynamically connected until the state period ends, so it is necessary to optimize the construction of the ICNS.

**Figure 3:**
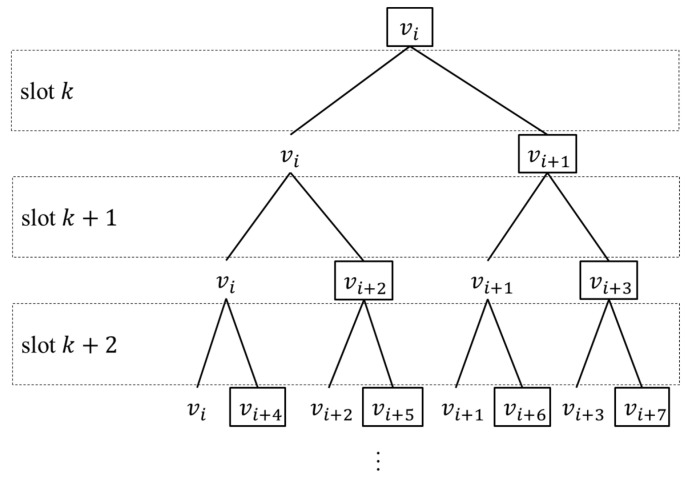
Binary tree showing dynamic paths.

We can prove that there is no plan to make ICNS dynamically connected during any slot. The binary tree in Figure 3 shows all dynamic paths that are from $v_{i}$. Nodes of this binary tree represent satellites. A parent node’s left child node is itself, and its right child node is the satellite with which it establishes an ISL during the corresponding slot. Satellites in different black rectangles are also different (their numbers are fictitious), which means each satellite cannot establish an ISL with satellites that it has connected with and their link objects. Under the circumstances, the binary tree can cover all |V| satellites when it has had $\log_{2}|V|$ levels, which means the maximum temporal length of dynamic paths from $v_{i}$ is $\log_{2}|V|$. Nevertheless, the link plan in Figure 3 is highly impracticable because it does not consider those dynamic paths from another satellite. If some satellites established an ISL with one of the link objects of its link objects, the maximum temporal length of dynamic paths from $v_{i}$ may increase to at least $1+\log_{2}|V|$. Therefore, $\log_{2}|V|$ is the ideal minimum of the maximum temporal length of dynamic paths. Hence, the ICNS will spend at least $\log_{2}|V|$ slots dynamically connected to slot k, no matter what k is. However, if $k>\Gamma -\log_{2}|V|$, certain dynamic paths will not finish before the state period ends, which means that the ICNS will never be dynamically connected from slot k to the last slot of the state period.

### 4.3. Dynamic-Connectivity-Oriented Construction Method of ICNS

The dynamic-connectivity-oriented construction method of ICNS is illustrated in Algorithm 2, which is also the process of constructing an ICNS in this study. 

We employed the Jaccard distance and the blacklist in Algorithm 2. The Jaccard distance is a measure of the similarity of two sets. For two non-empty finite sets, Y and Z, their Jaccard coefficient is the ratio of the cardinality of their intersection to the cardinality of their union [25], as shown in Equation (16):

(16) $$J\left(Y,Z\right)=\frac{\left|Y\cap Z\right|}{\left|Y\cup Z\right|}$$

The greater J(Y,Z) is, the more similar Y and Z are. The blacklist is the set shown in Equation (17):

(17) $$B=\{{\tilde{e}}_{ij}|{\tilde{e}}_{ij}\in \underset{>\kappa }{\underset{︸}{{\bar{M}}_{\zeta }\cap \cdots \cap {\bar{M}}_{\rho }}}\}$$

where κ is the maximum number of times that one CLC is permitted to repeat. The blacklist collects those CLCs that have occurred frequently in selected CCLCs. Any CCLC with even one element of the blacklist should be removed from the subset of all CCLCs ${ℳ}_{k}$ obtained by Algorithm 1.
**Algorithm 2.** Dynamic-connectivity-oriented construction method of ICNS.**Input**: $A_{k}$$,\;D_{k}$$,\;W_{k}$$,\;S=\{{\bar{M}}_{1},\cdots ,{\bar{M}}_{k-1}\}$$,\;\hat{E}=E_{1}\cup \cdots \cup E_{k-1}$ **Output**$:\;E_{k}$ 1:  Establish three matrices: $A^{\prime }={\left({a^{\prime }}_{ij}\right)}_{\Theta \times \Theta }$$,\;D^{\prime }={\left({d^{\prime }}_{ij}\right)}_{\Theta \times \Theta }$ and $W^{\prime }={\left({w^{\prime }}_{ij}\right)}_{\Theta \times \Theta }$$;\;A^{\prime }\leftarrow A_{k}$
   , $D^{\prime }\leftarrow D_{k}$, $W^{\prime }\leftarrow W_{k}$
2:  Obtain ${{ℳ}^{\prime }}_{k}$ based on $A^{\prime }$ using Algorithm 1  3:  Obtain the MSE-CCLC in ${{ℳ}^{\prime }}_{k}$, denoted by ${\bar{M}}_{k}$, based on $D^{\prime }$ and $W^{\prime }$
 4:  **if**
${\bar{M}}_{k}\notin S$
**then**
 5:    $E_{k}=\{e_{\lambda \mu }|{\tilde{e}}_{\lambda \mu }\in {\bar{M}}_{k}\}$
 6:  **else**
 7:     For each $e_{ij}\in \hat{E}$$:\;{a^{\prime }}_{ij}\leftarrow 0$$,\;{a^{\prime }}_{ji}\leftarrow 0$$,\;{w^{\prime }}_{ij}\leftarrow 0$$,\;{w^{\prime }}_{ji}\leftarrow 0$$,\;{d^{\prime }}_{ij}\leftarrow {d^{\prime }}_{ij}-1$,      ${d^{\prime }}_{ji}\leftarrow {d^{\prime }}_{ji}-1$
 8:     Obtain ${{ℳ}^{\prime }}_{k}$ based on new $A^{\prime }$ using Algorithm 1  9:     **if**
${{ℳ}^{\prime }}_{k}\neq \emptyset$
**do**
 10:   Obtain the MSE-CCLC in ${{ℳ}^{\prime }}_{k}$, denoted by ${\bar{M}}_{k}$, based on new $D^{\prime }$ and new $W^{\prime }$
 11:    $E_{k}=\{e_{\lambda \mu }|{\tilde{e}}_{\lambda \mu }\in {\bar{M}}_{k}\}$$,\;S\leftarrow S\cup \{{\bar{M}}_{k}\}$$,\;\hat{E}\leftarrow \hat{E}\cup E_{k}$
12:  **else**
 13:   Obtain ${ℳ}_{k}$ based on $A_{k}$ using Algorithm 1  14:   ${ℳ}_{k}\leftarrow {ℳ}_{k}-({ℳ}_{k}\cap S)$
15:   Establish the matrix $Y={\left(y_{\varepsilon \tau }\right)}_{\left|{ℳ}_{k}|\times |S\right|}$, where $y_{\varepsilon \tau }=J(M_{k}(\varepsilon ),E_{\tau })$$,\;1\leq \varepsilon \leq |{ℳ}_{k}|,$
      1≤τ≤k−1
16:   Establish the sequence: ${\bar{y}}_{1},\cdots ,{\bar{y}}_{\varepsilon },\cdots ,{\bar{y}}_{\left|{ℳ}_{k}\right|}$, where ${\bar{y}}_{\varepsilon }=\frac{1}{k-1}\sum_{\tau =1}^{k-1}y_{\varepsilon \tau }$ ; sort the       sequence from small to large, which creates the new sequence:        ${y^{\prime }}_{1},\cdots ,{y^{\prime }}_{\sigma },\cdots ,{y^{\prime }}_{\left|{ℳ}_{k}\right|}$; define the mapping f(⋅) and let $f({y^{\prime }}_{\sigma })=\varepsilon$
 17:   **for**
σ=1
**to**
$\left|{ℳ}_{k}\right|$
**do**
 18:    **if**
$M_{k}[f({y^{\prime }}_{\sigma })]\cap B\neq \emptyset$
**and**
$M_{k}[f({y^{\prime }}_{\sigma })]\notin S$
**then**
 19:     $E_{k}=\{e_{\lambda \mu }|{\tilde{e}}_{\lambda \mu }\in M_{k}[f({y^{\prime }}_{\sigma })]\}$$,\;S\leftarrow S\cup \{M_{k}[f({y^{\prime }}_{\sigma })]\}$$,\;\hat{E}\leftarrow \hat{E}\cup E_{k}$
 20:        **break**

21:    **end if**

22:   **end for**

23:   **end if**

24: **end if**


There are two ways to avoid a satellite from constantly establishing the ISL with the same satellite in Algorithm 2. The first is to modify three fundamental matrices of ${\tilde{G}}_{k}$ and to forbid those selected CLCs from being selected again, as line 6-11 introduces; it is able to make satellites establish ISLs with different satellites during different slots. The second is to select the CCLC that is most dissimilar to those selected CCLCs and does not have any element of the blacklist, as line 12–23 introduces; it allows satellites to establish ISLs with their previous link objects but the numbers of repeats cannot exceed the limit in the blacklist, i.e., κ in (17). If $|{\tilde{O}}_{i}|\geq \Gamma$, the first method is applied; otherwise, both methods are used.

## 5. Simulation Analyses

### 5.1. Simulation Analyses for the Construction of an ICNS

The satellite swarm consists of 30 satellites [26]:

8. Three geosynchronous orbit satellites are at an altitude of 35,786 km and longitude of 80 °E, 110.5 °E, and 140 °E, respectively, and they are numbered from 1 to 3;
9. Three inclined geosynchronous orbit satellites are spaced with 120 ° phase intervals at an attitude of 35,786 km and inclination of 55 °, and they are numbered from 4 to 6;
10. Twenty-four medium earth orbit satellites constitute a 24/3/1 walker constellation with an altitude of 21,528 km and an inclination of 55 °, and they are numbered from 7 to 30.

The satellite constellation above is also used in [2,3,4,5,6,7].

The length of the task time interval was set as 4 min from 17:17:00 to 17:21:00 on 16 August 2015. The state period could be set to 1 min or 2 min according to the visibility between satellites during the task time interval. Each slot lasted for 3 s, which is the rule of thumb introduced by the authors in [27]. Therefore, we designed four simulation groups, as shown in Table 1. Simulation group 2 and simulation group 4 used the method in Section 3, which did not improve the construction of ICNS. On the contrary, simulation group 1 and simulation group 3 used the method in Section 4 to improve the construction of the ICNS. The proposed methods were implemented in Java.

**Figure 4:**
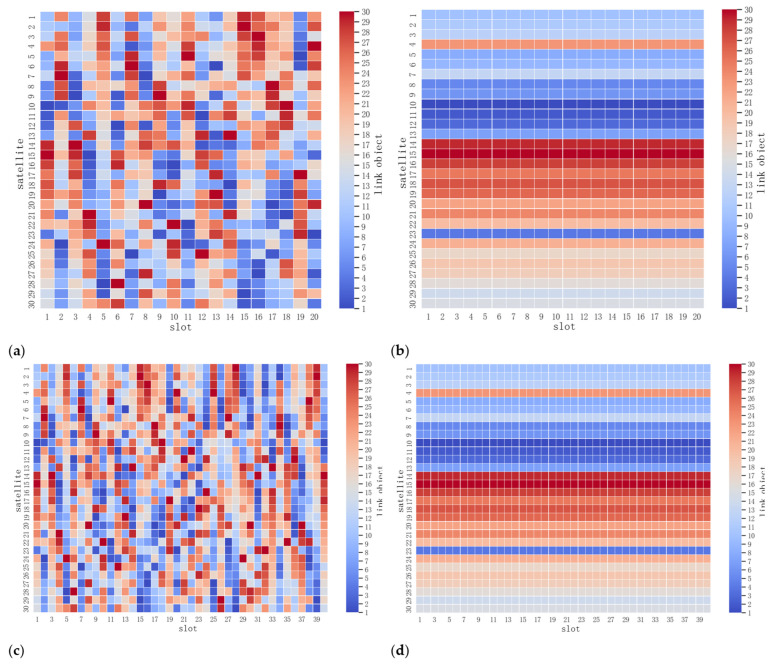
Results of the constructions of ICNS: (**a**) Simulation group 1; (**b**) Simulation group 2; (**c**) Simulation group 3; (**d**) Simulation group 4.

The ICNSs that were constructed in four simulation groups in Table 1 are illustrated in Figure 4. As the PLN for each slot in each simulation group had a perfect matching, there was $\theta =\frac{1}{2}\Theta =15$ in the ICNS for each slot. As Figure 4b,d show, each satellite in the ICNSs without improvement had an established ISL with the same satellite during the entire state period. These terrible situations have never happened in ICNSs with improvements, as is shown in Figure 4a,c. We only analyzed the results of simulation group 1 and simulation group 3, as the results of simulation group 2 and simulation group 4 were quite disappointing.

**Figure 5:**
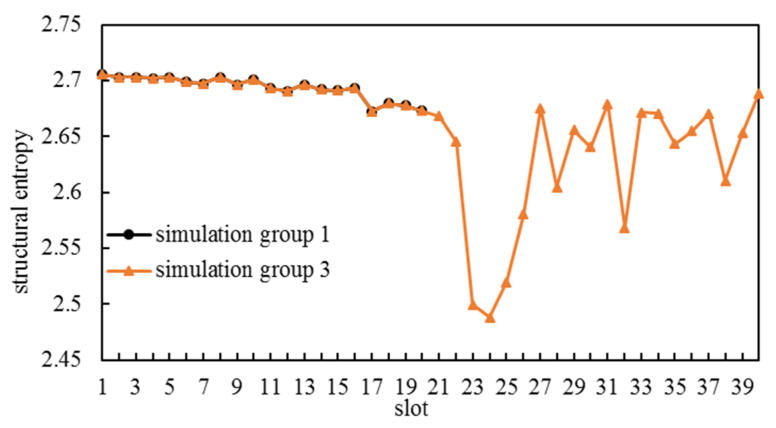
Structural entropies of CCLC within each slot.

The structural entropies of CCLCs that were selected to become edge sets of ICNSs are illustrated in Figure 5. The CCLC for each one of the first 20 slots in simulation group 3 had the same structural entropy as the CCLC for the corresponding slot in simulation group 1. As Figure 4b shows, there was ${\bar{M}}_{k}\in S$ from slot 2 to slot 24, and consequently, these CCLCs were selected using the first method of Algorithm 2. There was a downtrend in the structural entropies of these CCLCs. As more and more CLCs are selected over time and they cannot be selected anymore, CCLCs become less available. There was even one CCLC in ${{ℳ}^{\prime }}_{k}$ that was obtained by line 8 of Algorithm 2, and its structural entropy was minimal.

There was ${{ℳ}^{\prime }}_{k}=\emptyset$ from slot 25 to slot 40, during which CCLCs were selected using the second method of Algorithm 2. Under the circumstance, the structural entropy could not be used as a selection criterion so the structural entropies of these CCLCs fluctuated within a wider range.

**Figure 6:**
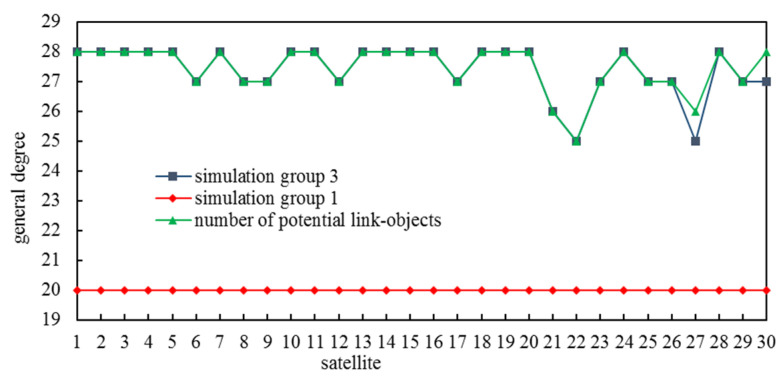
General degree distribution.

There were 300 ISLs established during 20 slots in simulation group 1 and 412 ISLs established during 40 slots in simulation group 3. The general degrees and the numbers of potential link objects of all satellites are shown in Figure 6. In simulation group 1, every satellite’s general degree equaled the number of slots. In simulation group 3, there were 28 satellites whose difference between numbers of potential link objects and general degree was zero, with 2 satellites whose difference was 1. Obviously, our method fully exploited ISL resources to construct an ICNS.

### 5.2. Simulation Analyses of Dynamic Connection of ICNS

We designed simulations on the dynamic connection of ICNS, which was based on the results of simulation group 1 and simulation group 3 in Section 5.1. We planned dynamic paths from each satellite to all other satellites during each slot, that is, there was a simulation case of $DP(v_{i},v_{j},k)$ for each possible combination of a source node $v_{i}\in V$, a destination node $v_{j}\in V$ ($v_{i}\neq v_{j}$), and a plan slot k (1≤k≤Γ). Then, we computed DCD for each slot according to the results of planning dynamic paths. If $DP(v_{i},v_{j},k)$ did not exist, DCD for slot k was 0.

**Figure 7:**
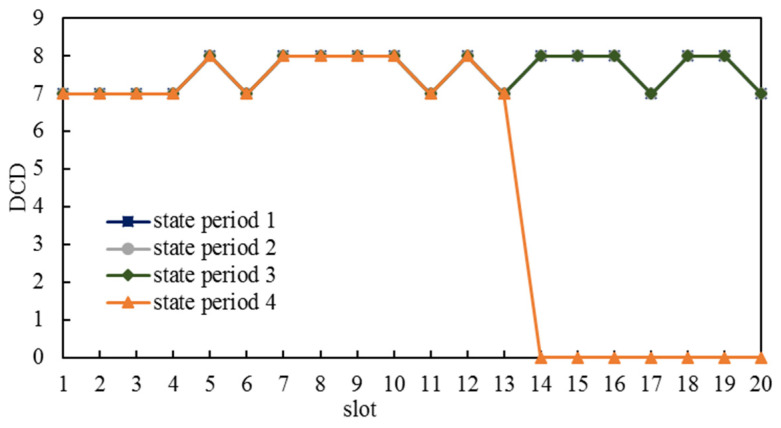
Dynamic-connect durations in the simulation group 1.

The results of simulation group 1 are shown in Figure 7.

**Figure 8:**
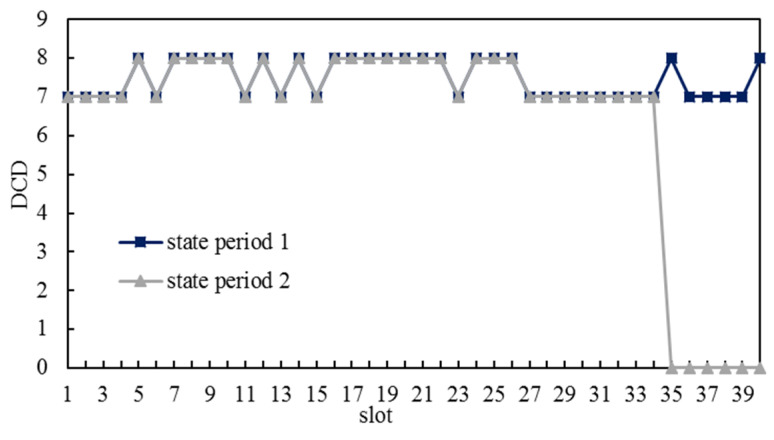
Durations of dynamic connectivity in simulation group 3.

As the blue, grey, and green lines in Figure 7 show, DCDs for the same slot in state period 1, state period 2, and state period 3 were the same. However, in state period 4, some S&Ds did not have available dynamic paths from slot 14 to slot 20, and correspondingly, the ICNSs were not able to be dynamically connected from slot 14 onwards. The reason for this phenomenon was that a dynamic path whose plan slot was at the back of a state period may have finished until the next state period. For S&Ds that had planned dynamic paths from slot 14, if they were in the first three state periods, their dynamic paths may have finished in the next state period, which is still within the task time interval; nevertheless, if they were in the last state period, their dynamic paths may not have finished at the end of task time interval, so these dynamic paths are believed to have been non-existent in the task time interval. The results of simulation group 3 are shown in Figure 8, which are similar to simulation group 1 and will not be introduced in this paper.

### 5.3. Impact of k-Value on ICNS

We let κ=2 in (17), in order to conduct simulations in Section 5.1, which meant those CLCs that occurred more than two times in selected CCLCs needed to be added to the blacklist. The impact of *κ*-values on ICNS is studied in this section. As Figure 5 shows, only the first method of Algorithm 2 was adopted to select the CCLC in simulation group 1, which did not need the blacklist; thus, we focused on simulation group 3. 

Firstly, we constructed ICNSs through Algorithm 2 with different *κ*-values. Secondly, we conducted simulations on the dynamic connectivity of these ICNSs, as detailed in Section 5.2. Finally, we analyzed the simulation results. The range of *κ*-values was set to [0,5].

**Figure 9:**
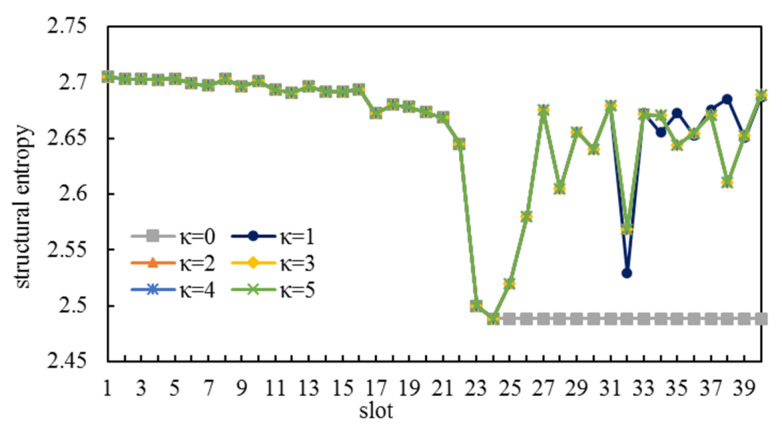
Structural entropies of CCLC under different *κ-*values.

We did not demonstrate the constructed ICNSs, as shown in Figure 4, due to limited space, while we adopted structural entropies to show the corresponding CCLCs, as shown in Figure 9. We can draw the following three conclusions:

The result of κ=0 was the worst. No new CCLC was obtained by Algorithm 2 from slot 25 onwards (CCLCs from slot 25 to slot 40 were all the same as the CCLC for slot 24 in Figure 9), which means that the ICNS cannot be dynamically connected from slot 25. Moreover, κ=0 means that any two satellites have to establish an ISL only once. A satellite will have no available satellite to establish an ISL with if the number of the slot is not smaller than the number of its potential link objects. 

If κ≥2, the CCLCs for the same slot are identical, even though they are under different κ-values. Therefore, different κ-values may not lead to different ICNSs and it is pointless to set a greater κ-value. 

If κ≥1, the CCLCs for each one of the first 31 slots under different κ-values are identical. Thus, not all CCLCs that are selected using the second method of Algorithm 2 are affected by κ-values. 

Then, we compared and analyzed several properties with respect to the dynamic-connection of ICNSs for κ=1 and κ=2.

**Figure 10:**
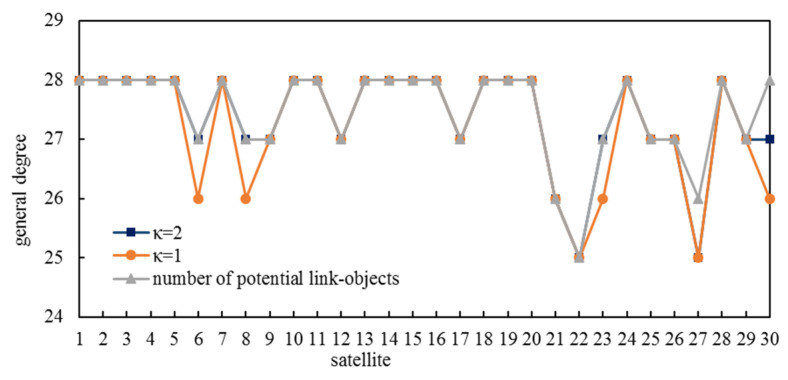
General degree of all satellites under different *κ*-values.

Figure 10 shows the general degree of all satellites under these two *κ*-values. There were 25 satellites whose general degree equaled the number of potential link objects if κ=1, which was less than 28 if κ=2.

Table 2 illustrates other dynamic connectivity properties. Since ICNS could not remain dynamically connected during the whole state period 2, we only introduced the results of state period 1. Obviously, ICNS under κ=2 had better dynamic connectivity properties than ICNS under κ=1, and that is why we adopted κ=2 in Section 5.1.

### 5.4. Comparisons with the Benchmark Methods

In this section, the performance of the proposed method was compared with three benchmark methods proposed by the authors in [4,28,29]. In order to assign the ISLs in GNSS, the authors in [28] used the discrete differential evolution (DDE); the authors in [29] used the genetic algorithm (GA); and the authors in [4] used RWM and RWMWE, as mentioned in Section 1. As these benchmark methods concentrate on GNSS, not all of the indicators used were fit for our study. There were two available indicators. One was the average number of ISLs for all satellites among all slots in the state period; the other was the average path temporal length (APTL). The satellites that are invisible to the ground station were defined as overseas satellites, and the others were called domestic satellites. APTL is the average value for all overseas satellites in all state periods, which can be obtained by:

(18) $$APTL=\sum_{i=1}^{S_{o}}\sum_{k=1}^{K}PTL_{ik}$$

where $PTL_{ik}$ is equivalent to the length temporal of the shortest dynamic path from an overseas satellite i at slot k to any domestic satellite at subsequent slots [4]; $S_{o}$ is the number of overseas satellites; and k is the number of slots.

As the satellite constellation introduced in Section 5.1 was identical to the simulated satellite constellations in [4,28,29], we just needed to execute a simulation with the same parameters. The main parameters of the simulation setup are shown in Table 3.

Table 4 illustrates the comparison results of the proposed method and three benchmark methods for 10,080 state periods. The average number of ISLs were calculated for all satellites and 10,080 state periods. The proposed method obtained the minimum average number of ISLs, which was 19.91. The APTL of the proposed method was 1.33, which was the second smallest APTL.

## 6. Conclusions

This paper describes how a satellite swarm creates an ICNS with the theory of complex networks. We proposed the community-based construct method of ICNS and its improvement method. The results regarding constructing ICNSs have proven that it is necessary to improve the construct method of an ICNS, which helps an ICNS to maintain dynamic connectivity continuously. The results of dynamic connectivity simulations of an ICNS indicate that an ICNS cannot be dynamically connected in the last few slots of the last state period. Therefore, a satellite swarm should fulfill its task as soon as possible; otherwise, poor communication may occur at the end of the task time interval.

The proposed method is capable of constructing an ICNS with a good dynamic connection quickly. It has many advantages, such as being a simple model, and has good flexibility, adaptability, and expansibility. In fact, the ICNS constructed by our method can be further improved with some intelligent optimization methods and reinforced learning algorithms in order to achieve a certain object.

Besides community, path, and connectivity, other concepts and properties of complex networks may also be adopted in an ICNS, such as invulnerability, synchronization, and traffic dynamics, which will be the subject of our future work.

## Figures and Tables

**Table 1:** Introductions of simulation groups.

Simulation Group Number	Improved or Not	Number of Slots
1	Yes	20
2	No	20
3	Yes	40
4	No	40

**Table 2:** Dynamic connectivity properties under different values of *κ.*

*k*-Value	Number of ISLs	Average Spatial Length of Dynamic Paths	Average Temporal Length of Dynamic Paths	Average DCD
1	409	2.758	4.556	7.7
2	411	2.731	4.494	7.475

**Table 3:** Main parameters of the simulation setup.

Parameter	Value
Task time interval	10,080 min (1 week)
State period	1 min
Slot	3 s

**Table 4:** Results of the different methods.

Method	Average Number of ISLs	APTL
The proposed method	19.92	1.33
RWM	17.28	1.49
RWMWE	14.85	1.15
DDE	12.23	2.12
GA	9.63	1.38

## Data Availability

Not applicable.
